# Successful Outcome of Low-Dose S-1 Used to Treat Buccal Squamous Cell Carcinoma

**DOI:** 10.1155/2017/4537631

**Published:** 2017-07-18

**Authors:** Kazuyuki Yusa, Hideyuki Yamanouchi, Ayako Sugano, Mitsuyoshi Iino

**Affiliations:** ^1^Department of Dentistry, Oral and Maxillofacial, Plastic and Reconstructive Surgery, Yamagata University Faculty of Medicine, Yamagata, Japan; ^2^Department of Dentistry and Oral Surgery, Shinjo Tokushukai Hospital, Yamagata, Japan

## Abstract

This case report describes an 86-year-old woman with dormant right buccal squamous cell carcinoma who was able to maintain a reasonable quality of life after being treated with oral low-dose S-1 (80 mg/day). The treatment regimen started in April 2014 and consisted of two weeks of S-1 followed by a one-week interval. The patient remains on this regimen while maintaining her quality of life and she has been under follow-up as an outpatient for 36 months. The outcomes for this patient indicated that low-dose S-1 is a valid anticancer therapy that may help maintain quality of life for some patients with incurable or dormant cancers.

## 1. Introduction

The destruction of normal tissues by head and neck cancers often results in dysfunction, and postoperative sequelae such as speech disorders, pain, depression, dysphagia, and xerostomia are common and often significant [1, 2]. Hence, the approach to treating cancers of the head and neck should be selected depending on the age, general health status, and prognosis of patients, as well as their comprehension. Fundamental treatment strategies such as tumor resection or chemoradiotherapy are not usually the first choice for treating elderly patients with head and neck carcinoma. Also, whether intensive chemotherapy using platinum drugs, taxanes, methotrexate, or bleomycin can improve the quality of life of such patients is doubtful [3, 4].

Although its effectiveness as chemotherapy for head and neck carcinoma has not been fully demonstrated, S-1 might be safe and effective for treating elderly patients with head and neck carcinoma. This case report describes an elderly patient with dormant buccal squamous cell carcinoma who could maintain a reasonable quality of life with long-term low-dose S-1.

## 2. Case Presentation

**Figure 1:**
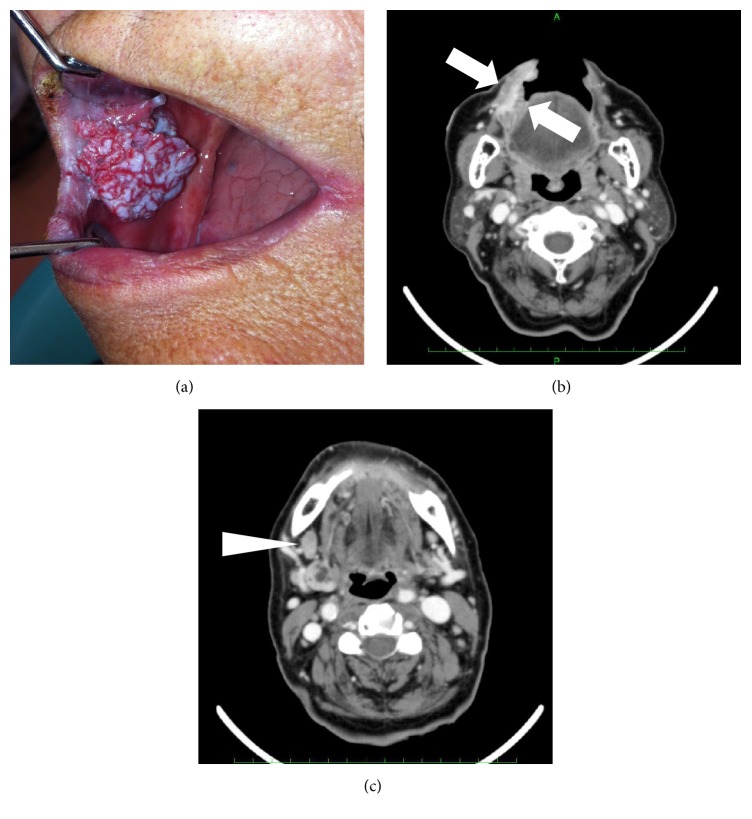
*Intraoral findings at initial presentation*. Ulcerated lesion in the right buccal area (a). CT images in the right buccal area show an enhanced lesion ((b), arrow) and enlarged submandibular lymph node ((c), arrow head).

A family dentist referred an 86-year-old woman to us in March 2014 with a complaint of contact pain in the right buccal mucosa. Her medical history revealed hyperlipidemia and diabetes mellitus, which were controlled with medication. She had a gastric carcinoma that was resected in 2011, and she has remained free of postoperative recurrence and lymph node metastasis. She denied difficulty with swallowing, chewing, speech, and breathing. The findings of a general physical examination were unremarkable. An intraoral examination showed an ulcerated mass around the right buccal mucosa at a right angle to the mouth (Figure 1(a)), and CT imaging revealed a 31 × 18 mm enhanced lesion around the right buccal region (Figure 1(b)) and an enlarged submandibular lymph node (Figure 1(c)). A biopsy confirmed the lesion as well-differentiated squamous cell carcinoma classified as T2N1M0, stage III.

Neither the patient nor her family wished to consider radical therapy such as tumor resection, considering her advanced age. Therefore, we obtained written, informed consent from the patient and her family for oral administration of low-dose S-1 (80 mg/day) starting in April 2014. The regimen consisted of two weeks of S-1 therapy followed by a one-week interval. This regimen is ongoing and she has remained free of adverse reactions defined in the National Cancer Institute Common Toxicity Criteria version 4.0 toxicity scale.

**Figure 2:**
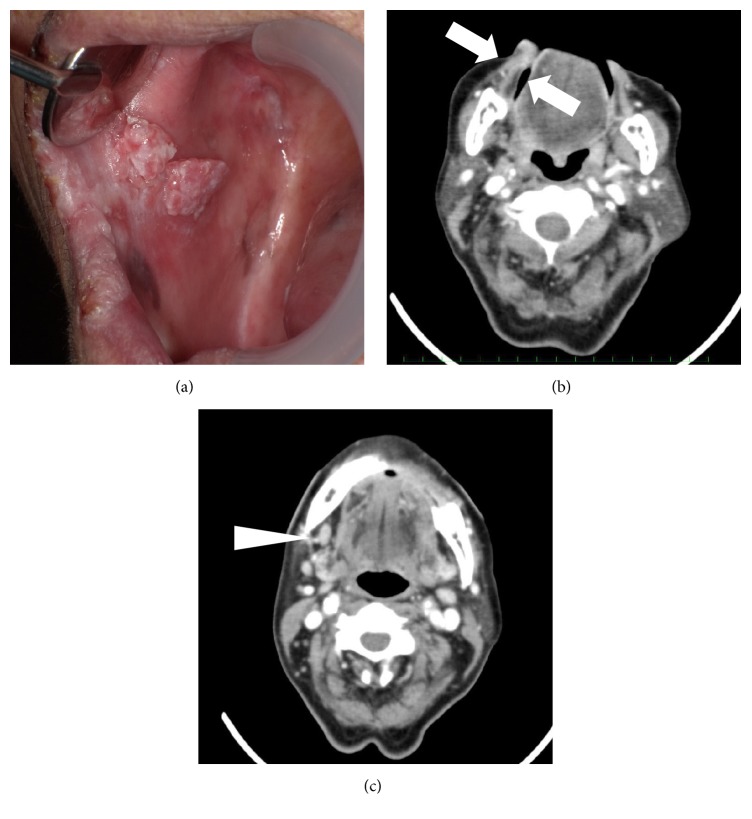
*Intraoral findings at most recent follow-up*. Intraoral findings (a) and CT images of the right buccal region (b, arrow) and submandibular lymph node (c, arrow head).

Figure 2 shows that the size of the tumor had decreased to 12 × 6 mm, both clinically (Figure 2(a)) and radiographically (Figure 2(b)) and that the size of the metastatic submandibular lymph node had also decreased (Figure 2(c)) at the time of the last follow-up. The patient has survived for 36 months since she was initially referred to us, and her quality of life has not decreased.

## 3. Discussion

S-1 is an oral anticancer agent containing tegafur, a prodrug of 5-fluorouracil (5-FU), and two modulators. One is 5-chloro-2,4-dihydroxypyridine, which causes prolonged retention of 5-FU in the bloodstream and enhances the pharmacological actions of 5-FU by competitively inhibiting its degradation. The other is potassium oxonate, which localizes in the mucosa of the gastrointestinal tract after oral administration and alleviates gastrointestinal toxicities induced by 5-FU [5, 6]. S-1 monotherapy is considered a second- or third-line chemotherapy for unresectable cancer [7]. Although response rates are modest, S-1 monotherapy is safe and well tolerated by elderly patients [8], and it can be administered over the long term in the outpatient setting. Thus, patients can undergo S-1 chemotherapy while maintaining a reasonable quality of life. The original S-1 regimen comprised four weeks of chemotherapy followed by two weeks of rest. However, Itoi et al. [9] reported that a regimen comprising two weeks of S-1 followed by one week of rest is less likely to be interrupted by side effects and is safer for outpatients.

Some studies of models have found that continuous low-dose chemotherapy can cause the long-term suppression of tumor proliferation in vitro and in vivo and that one of the action mechanisms involves antiangiogenic effects [10, 11]. Recently, much attention has been focused on metronomic chemotherapy; some successful drug combinations for several cancers have been reported [12]. Allegrini et al. [13] reported metronomic chemotherapy regimen including UFT plus cyclophosphamide (CTX) and celecoxib (CXB) in patients with advanced refractory gastrointestinal cancers. The results of this study suggested a possible antitumor activity of this metronomic regimen, comparable with those observed in the same setting patients treated with other chemotherapy. Tumor vasculatures instead of tumor cells were main target of these chemotherapy regimens, and the same mechanisms might have been involved in the outcome for our patient.

Low-dose S-1 chemotherapy for elderly patients with head and neck carcinoma appears to be safe in the outpatient setting and might be an effective strategy with which to maintain the quality of life of patients who have incurable and dormant tumors.
